# Supplementary figures and images for: Deep learning for classification of pediatric chest radiographs by WHO’s standardized methodology
Source: PLoS One. 2021 Jun 21;16(6):e0253239. doi: 10.1371/journal.pone.0253239 (PMC8216551; doi:10.1371/journal.pone.0253239)

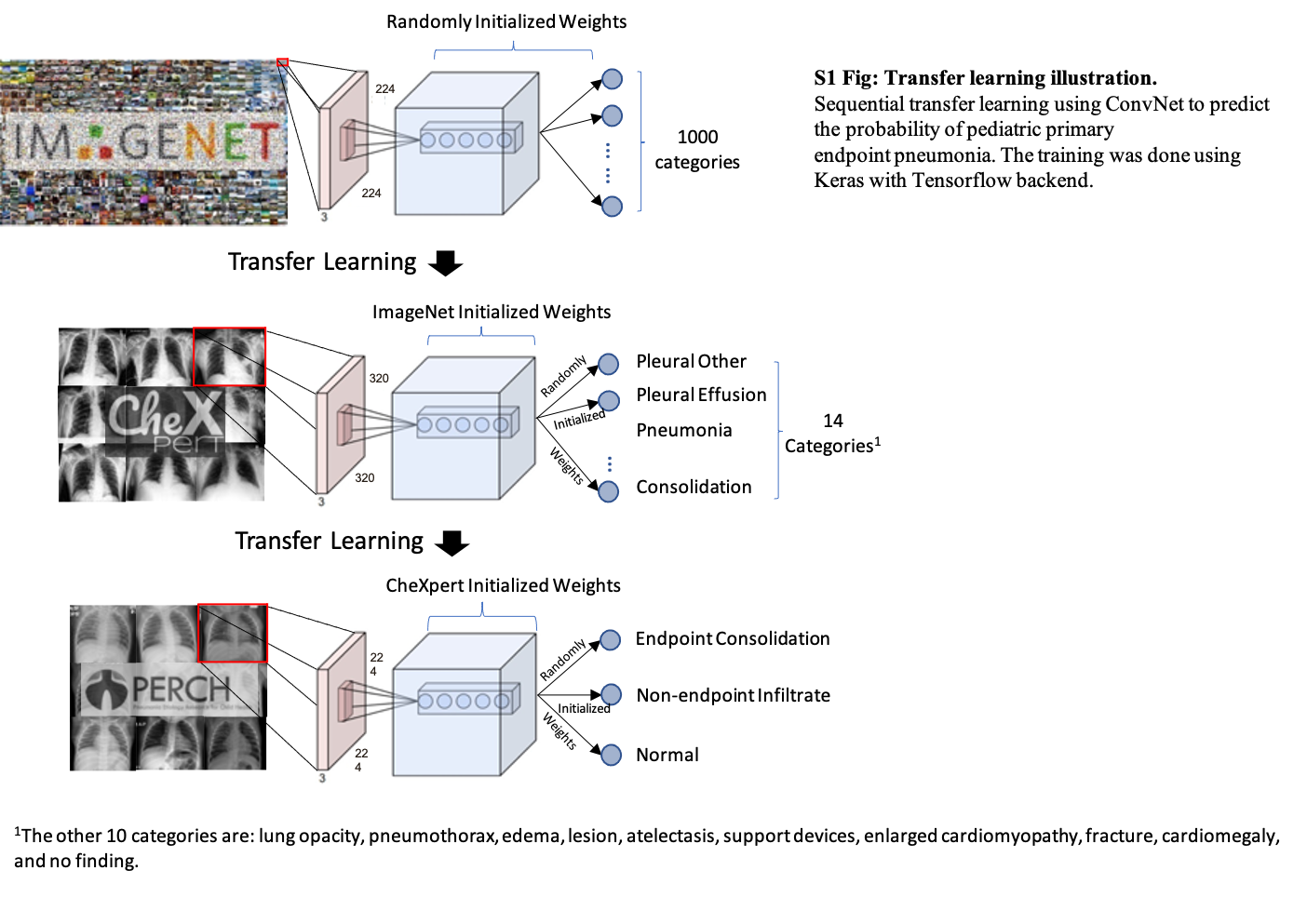

Supplement: S1 Fig — (PNG) [file pone.0253239.s001.png]

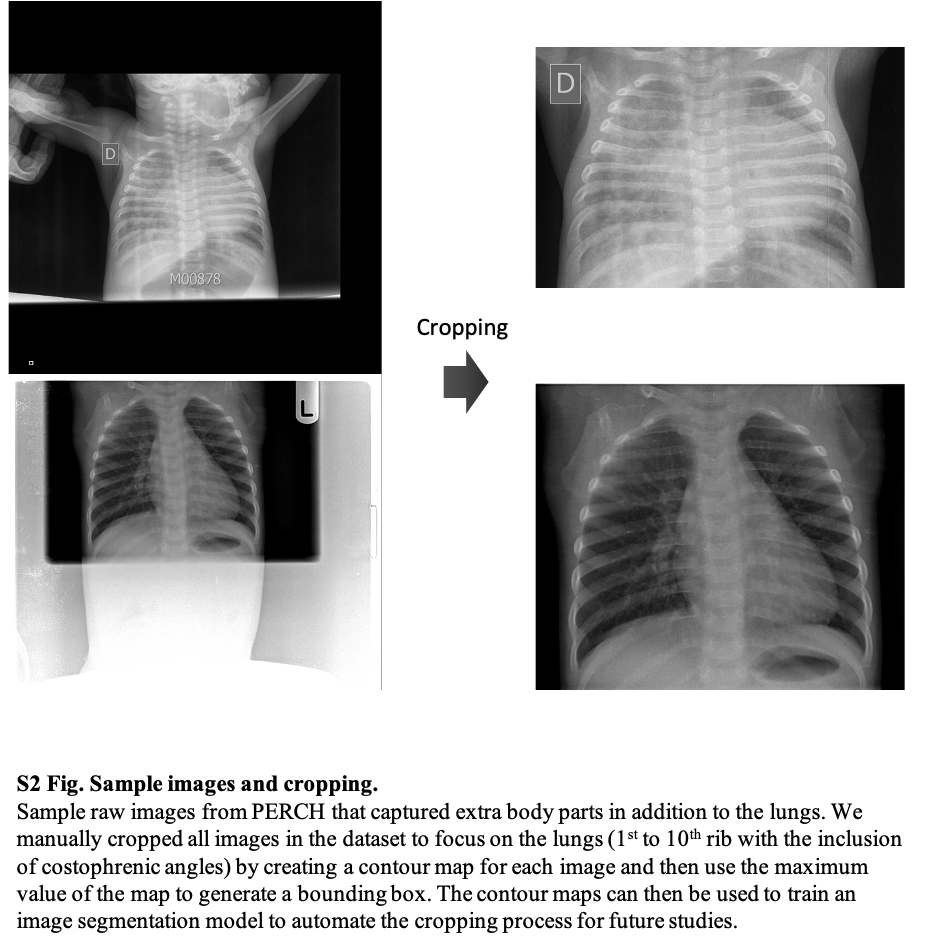

Supplement: S2 Fig — (PNG) [file pone.0253239.s002.png]

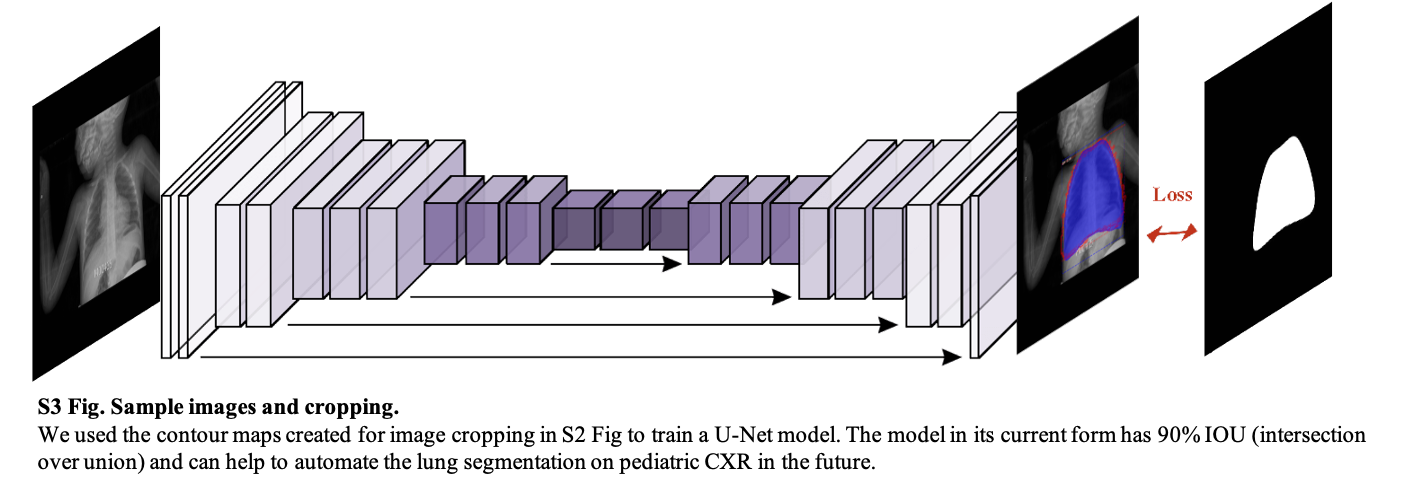

Supplement: S3 Fig — (PNG) [file pone.0253239.s003.png]

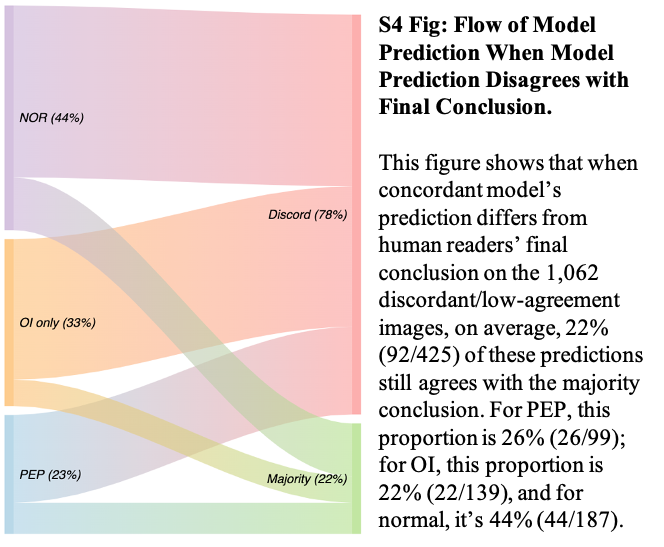

Supplement: S4 Fig — (PNG) [file pone.0253239.s004.png]
