# Supplementary material for: Deep learning for classification of pediatric chest radiographs by WHO’s standardized methodology
Source: PLoS One. 2021 Jun 21;16(6):e0253239. doi: 10.1371/journal.pone.0253239 (PMC8216551; doi:10.1371/journal.pone.0253239)
Supplement: S1 Table — (DOCX) [file pone.0253239.s005.docx]

**S1 Table**. AUROC scores (averaged across 10-folds) on the validation set, and WHO test set of the models trained on uncropped PERCH images

|  | PERCH Validation | WHO All | WHO-Original | | | WHO-CRES |
| --- | --- | --- | --- | --- | --- | --- |
| Category | (n=345) | (N=410) | High^a^ (n=120) | Low^b^ (n=88) | High + Low (n=208) | High (n=203) |
| Primary Endpoint Pneumonia | 0.909 (0.898,0.920) | 0.967 (0.962,0.972) | 0.981 (0.975,0.986) | 0.837 (0.808,0.865) | 0.933 (0.922,0.943) | 0.994 (0.992,0.996) |
| Other Infiltrates | 0.753 (0.735,0.771) | 0.855 (0.839,0.870) | 0.950 (0.935,0.966) | 0.700 (0.661,0.732) | 0.855 (0.839,0.870) | 0.916 (0.897,0.936) |
| Normal | 0.878 (0.867,0.889) | 0.940 (0.933,0.947) | 0.933 (0.921,0.946) | 0.740 (0.704,0.776) | 0.940 (0.933,0.947) | 0.969 (0.962,0.977) |
| **^a^** Images with high inter-observer agreement (i.e. ≥66% of readers agree on the label for the image)  ^b^ Images with low inter-observer agreement (i.e. <66% of readers agree on the label for the image) | | | | | | |
