# Supplementary material for: Deep learning for classification of pediatric chest radiographs by WHO’s standardized methodology
Source: PLoS One. 2021 Jun 21;16(6):e0253239. doi: 10.1371/journal.pone.0253239 (PMC8216551; doi:10.1371/journal.pone.0253239)
Supplement: S2 Table — (DOCX) [file pone.0253239.s006.docx]

**S2 Table**. Matrix of “Concordant Model” Predictions by Final and Majority Conclusions

| 1. *(N=1,062)* | | | |
| --- | --- | --- | --- |
| Final conclusion | Model prediction | | |
|  | Primary-endpoint | Other-infiltrates | Normal |
| Primary-endpoint | 257 | 78 | 54 |
| Other-infiltrates | 74 | 149 | 133 |
| Normal | 25 | 61 | 231 |
| *Kappa = 0.40*  2. *(N=655)* | |  |  |
| Majority conclusion | Model prediction | | |
|  | Primary-endpoint | Other-infiltrates | Normal |
| Primary-endpoint | 227 | 50 | 36 |
| Other-infiltrates | 21 | 76 | 52 |
| Normal | 14 | 33 | 146 |
| *Kappa = 0.33* | |  |  |
| 3*. (N=655)* |  |  |  |
| Majority conclusion | Final conclusion | | |
|  | Primary-endpoint | Other-infiltrates | Normal |
| Primary-endpoint | 258 | 45 | 10 |
| Other-infiltrates | 25 | 95 | 29 |
| Normal | 17 | 50 | 126 |

*Kappa = 0.41*

*Darkness of the cell color is proportional to the sample size in the cell.*

|  |  |  |  |
| --- | --- | --- | --- |

**S2 Table presents 3 comparisons:**

1. A comparison between the models’ predictions and the final conclusions of the arbitrator, where the model was trained on concordant (high-agreement) images only and tested on all discordant (low-agreement) images.
2. A comparison between the concordant model's prediction and the majority's conclusion (2 vs.1 vs.1) from 4 readings (2 readings from reviewers and 2 readings from arbitrators, where one reviewer agrees with one arbitrator on the same conclusion, while the other reviewer and arbitrator gave 2 different conclusions (prior to final stage of arbitration where the arbitrators discuss and agree on a final conclusion). A 2:2 tie where one reviewer agrees with one arbitrator on one conclusion and the other reviewer agrees with the other arbitrator on a different conclusion is considered to be a case that has no majority conclusion.
3. A comparison between final conclusions and majority conclusions from 4 readings.
